# Dietary habits and knee and shoulder injury incidence in adolescent male and female handball players: the Swedish Handball Cohort

**DOI:** 10.1136/bmjsem-2024-002332

**Published:** 2025-03-23

**Authors:** Clara Onell, Eva Skillgate, Pierre Côté, Markus Waldén, Henrik Källberg, Martin Hägglund, Klara Edlund, Anna Melin, Martin Asker

**Affiliations:** 1 Handball Research Group, Department of Health Promoting Science, Sophiahemmet University, Stockholm, Sweden; 2 Unit for Intervention and Implementation Research in Worker Health, Institute of Environmental Medicine, Karolinska Institutet, Stockholm, Sweden; 3 Institute for Disability and Rehabilitation Research and Faculty of Health Sciences, Ontario Tech University, Oshawa, Ontario, Canada; 4 Capio Ortho Center Skåne, Malmö, Sweden; 5 Sport Without Injury Programme (SWIPE), Department of Health, Medicine and Caring Sciences, Linköping University, Linköping, Sweden; 6 Department of Public Health, Analysis and Data Management, Public Health Agency of Sweden, Solna, Sweden; 7 Unit of Physiotherapy, Department of Health, Medicine and Caring Sciences, Linköping University, Linköping, Sweden; 8 Division of Psychology, Department of Clinical Neuroscience, Karolinska Institutet, Stockholm, Sweden; 9 Department of Sport Science, Linnaeus University, Växjö/Kalmar, Sweden, Swedish Olympic Research Fellow

**Keywords:** Epidemiology, Nutrition, Athlete, Adolescent, Sport

## Abstract

**Objectives:**

To assess the association between (1) dietary habits and knee/shoulder injury incidence in male and female adolescent handball players and (2) menstrual dysfunction and injury incidence in females.

**Methods:**

This study is based on seasons 2020–2022 of the Swedish Handball Cohort including 1144 participants (1703 player seasons) free from a substantial knee and shoulder injury. Participants self-reported meal frequency, meal timing, nutritional intake and menstrual function (season 2022/2023) at baseline. Weekly follow-ups throughout the season assessed training and matches, and substantial knee/shoulder injuries. Cox regression analyses estimated a hazard rate ratio (HRR) with the first event of a knee/shoulder injury (combined), with minutes of handball training and matches as the timescale.

**Results:**

In females, adjusted analyses generated an HRR for knee/shoulder injuries of 1.46 (95% CI 1.08, 1.98) for moderate-high nutritional quality compared with low quality and an HRR of 1.38 (95% CI 1.02, 1.86) for ≥2 unfavourable dietary habits compared with 1 unfavourable dietary habit. For poor meal timing, adjusted analyses generated an HRR of 1.20 (95% CI 0.90, 1.61) compared with adequate timing in females. In males, adjusted analyses generated an HRR of 1.23 (95% CI 0.69, 2.17) for low meal frequency and an HRR of 0.83 (95% CI 0.60, 1.15) for poor meal timing.

**Conclusions:**

In adolescent female handball players, moderate-high nutritional quality and ≥2 unfavourable dietary habits are associated with higher knee/shoulder injury incidence; whereas, no or unprecise associations were found for other dietary habits in females and males and for menstrual dysfunction in females.

**WHAT IS ALREADY KNOWN ON THIS TOPIC**

- Inadequate dietary habits such as low meal frequency and intake of low energy-dense foods are associated with injuries in adolescent athletes from weight-sensitive sports. Also, menstrual dysfunction has been found to be associated with injuries in female athletes. These associations have been less studied in team-sport athletes, such as in adolescent handball players at handball-profiled high schools.

**WHAT THIS STUDY ADDS**

- This study suggests that female adolescent handball players with a moderate-high nutritional quality (ie, moderate-high adherence to the Nordic Nutrition Recommendations) and ≥2 dietary habits not in accordance with sports nutrition recommendations have a higher rate of knee/shoulder injuries during the handball season, compared to females with a low nutritional quality and <2 dietary habits not in accordance with recommendations. For male adolescent handball players, the results are less certain.

**HOW THIS STUDY MIGHT AFFECT RESEARCH, PRACTICE OR POLICY**

- The findings may be valuable for athletes and coaches in order to implement nutritional strategies promoting health and performance. Highlighting the importance of a proper energy and nutrient intake in adolescent sports settings, where sports nutrition recommendations have precedence over national dietary guidelines, may bring sustainable health promotion for adolescent athletes.

## Background

Handball is a popular youth team sport alternating between offensive and defensive elements including rapid changes of direction, abrupt landings and repetitive throws. Given these characteristics, handball is one of the most injury-prone sports, where lower limb and shoulder injuries are most common.^1^ Studies on Swedish adolescent handball players show a 23% season prevalence^2^ of a substantial shoulder problem and a 32% one-year prevalence^3^ of a substantial knee problem, with a substantial problem being defined as a problem so severe that it impacts athletes’ training or performance. Sports injuries may have implications including persistent pain, discontinued sports participation, loss of social interactions, mental health problems and poor lifestyle behaviours.^4^,^6^

Insufficient energy and nutrient availability in adolescent athletes may compromise recovery and adaptation to training as well as increase the risk of injuries, poor bone health and delayed puberty.^7^ Imbalance between energy intake and expenditure with respect to training load may result in problematic (long-term/severe) low energy availability (LEA), manifested as relative energy deficiency in sports (REDs) with potential impact on metabolic, cardiovascular and psychological health.^8^ The prevalence of LEA and REDs indicators (ie, symptoms) is estimated to be 23%–80% in female athletes.^8^ Menstrual dysfunction is identified as a primary indicator of LEA, affecting an estimated 32% (range 8%–86%) of female athletes^9^ and is associated with impaired performance,^10 11^ bone stress injuries and musculoskeletal injuries in adolescent females.^11^,^13^

Handball players are recommended to have a sufficient intake of energy and nutrients to maintain health and performance. Yet, studies report that handball players, just like athletes in many other disciplines, fail to meet sports nutrition recommendations for energy and carbohydrates^14^,^16^ as well as micronutrients.^17^,^19^ Meal skipping^20^ and non-adherence to recommended intakes of fruits, vegetables and fish^21^ have been associated with injuries in adolescent athletes. In a cross-sectional study of 1040 adolescent handball players,^22^ we found that meal frequency of ≥4 meals per day and meal timing before and after training were in accordance with sports nutrition recommendations; whereas, adherence to general recommendations of fruits/vegetables intake of ≥500 grams per day and fish/seafood intake of ≥2 times per week was poor. Furthermore, reports on fewer daily meals and more supplementation of vitamins and minerals in adolescent female handball players compared with males^22^ indicate that females may have more restrictive dietary behaviours and, thus, may be at risk of problematic LEA and injuries.^13 20^

To the best of our knowledge, cohort studies investigating the association between dietary habits and injury incidence in adolescent handball players are lacking. Knowledge about potential risk factors for the most common and burdensome injuries in this population is warranted. Therefore, in this study, the primary aim was to assess the association between dietary habits and knee/shoulder injury incidence in adolescent male and female handball players. The secondary aim was to investigate the association between menstrual dysfunction and knee/shoulder injuries in adolescent female handball players.

## Methods

This study is based on data from the Swedish Handball Cohort (SHC), a cohort study with the overall aim to investigate risk factors and evaluate implementation of injury prevention exercise programmes in youth handball. Accredited handball-profiled high schools in Sweden were invited to participate. For the schools that accepted participation in the study, the responsible handball instructor distributed the study information to the players. The inclusion criterium in the SHC was enrolment to any of the handball-profiled high schools in Sweden that agreed to let their handball players be asked to participate in 2020–2022. There were no exclusion criteria. The web-based baseline survey and weekly follow-ups were administered electronically by a survey platform (lynes Technologies Sweden AB). Participants responded to the baseline web-survey in the beginning of the handball season and were monitored with web-based follow-ups every week on Sundays throughout the season, for approximately 8 months. Two reminders about responding to the follow-ups were sent through the lynes smartphone application as well as emails and short text messages (SMS). Participants who had not responded within 4 days were reminded by study personnel through a phone call.

### Study population

Thirty-six Swedish handball-profiled high schools with a total of 1991 potential participants were invited to participate in the SHC seasons 2020/2021, 2021/2022 and 2022/2023. Of those, 1251 adolescent handball players (63%) from 22 schools (61%) agreed to participate. A total of 107 participants with a substantial knee or shoulder injury at baseline were excluded, resulting in a sample of 1144 shoulder and knee injury-free participants (542 females, 603 males). Season 2020/2021 included 551 participants (271 females, 280 males), season 2021/2022 included 600 participants (280 females, 320 males) and season 2022/2023 included 552 participants (244 females, 308 males). A total of 467 participants were included in more than one season, resulting in 1703 player seasons. The average weekly follow-up rate during the three seasons was 75%.

### Baseline measurements

Participants responded to the baseline survey in the fall of 2020, and/or 2021, and/or 2022. The survey included questions about demographics, playing level, playing position, training and sleep habits, past and current injuries and dietary habits. Questions related to menstrual function were added for females in the baseline survey season 2022/2023. A summary of all exposures, outcomes, covariates and categorisation in the statistical analyses is presented in online supplemental table 1.

### Meal frequency

Daily meal frequency was reported as intake frequency of main meals and in-between snacks. Low meal frequency was dichotomised to reporting <4 meals per day.

### Meal timing

Meal timing was reported as having a main meal, a snack or no meal within 3 hours before and 1 hour after training, and consuming carbohydrate-rich foods and fluids (eg, gels/bars) during matches. Poor meal timing was dichotomised to reporting no main meal within recommended time spans before/after training, as well as reporting no carbohydrate intake during matches.

### Nutritional quality

A food frequency questionnaire (FFQ) developed by the Swedish Food Agency^23^ was used to assess the diet’s nutritional quality (NQ) as adherence to the Nordic Nutrition Recommendations (NNR). The habitual intake frequency of vegetables/legumes, fruits/berries, whole-grain bread, crispbread, fried potatoes/French fries, fish/seafood, sausage, chocolate/candy, pastries, high-fat cheese and sugar-sweetened beverages over the prior 12 months were reported from never or <1 time per month to ≥4 times per day. The intake frequency of each food item corresponds to an NQ score based on adherence to the NNR (online supplemental table 2). A sum score of each food item’s score generated an NQ index between 0 and 12 where scoring 0–4 corresponded to low NQ, 5–8 to moderate NQ and 9–12 to high NQ, according to the Swedish Food Agency.^23 24^ The reliability and criterion validity of the NQ index has been shown to be acceptable.^23^ A moderate-high NQ was dichotomised to an NQ score ≥5.

### Menstrual function

Menstrual function was assessed in females responding to the baseline survey in season 2022/2023 with items from the Low Energy Availability in Females Questionnaire (LEAF-Q)^25^ including time since last menstrual cycle and number of menstruations the past 12 months. In females reporting no use of hormonal contraceptives, menstrual dysfunction was dichotomised to reporting no menarche (primary amenorrhea), no menstrual bleeding for ≥3 consecutive months (secondary amenorrhea) or ≤8 menstruations the past 12 months (oligomenorrhea).^26^

### Follow-up measurements

The follow-ups were weekly web-based reports sent out from baseline until May in 2021, 2022 and 2023 including questions about training and match volume, injury and lost time from handball participation over the prior week, as well as the Oslo Sports Trauma Research Center Overuse Injury Questionnaire (OSTRC-O).^27^

### Knee/shoulder injuries

A Swedish version^28^ of the OSTRC-O was used to assess the outcome. The OSTRC-O evaluates injury consequences with respect to sports participation, training volume, sports performance and pain,^29^ assessed in participants reporting other than full participation. In this study, the outcome was the first event of a substantial knee/shoulder injury, defined as reporting having at least a moderate reduction in training volume or performance the past week due to a knee or shoulder problem (combined).

### Statistical analyses

A prevalence ratio with a 95% CI for dietary habit exposures by sex was calculated by dividing the prevalence of each risk dietary habit in females by the prevalence of the same habit in males. Crude and adjusted Cox proportional hazard regression models were built to assess the association between the exposures of low meal frequency, poor meal timing, moderate-high NQ, ≥2 unfavourable dietary habits (ie, low meal frequency, poor meal timing and/or moderate-high NQ) and menstrual dysfunction and the outcome knee/shoulder injuries. Associations were reported as a hazard rate ratio (HRR) with a corresponding 95% CI.

Assumptions for Cox regression analyses were checked with plots of the Schoenfeld and Martingale residuals indicating proportional hazards and no non-linearity or influential observations. Sensitivity analyses with adjustment for being included more than one season were performed stratified by sex. Total weekly minutes in handball training and matches were the underlying timescale, starting from the first week of follow-up throughout the handball season, where each participant had a cumulative exposure time at risk. Participants lost to follow-up after baseline were censored.

Since time at risk was based on amount of handball training and matches, those reporting other reasons than knee/shoulder injuries for not fully participating in handball the past week (eg, school break, other injury/illness) were not censored. Participants were assigned a unique player season identification number and handled as a ‘new’ participant in each season, if they participated more than one season. If re-entering as a new player season the next season, the participants were included again despite having an injury during the past season.

Potential confounding factors were chosen a priori based on clinical experience and scientific literature, considering their potential role as mediators or colliders, as illustrated in a directed acyclic graph (DAG) in online supplemental figure 1. All suggested covariates were included in the final model. Data management and statistical analyses were performed in R version 4.2.1 (2022-06-23 UCRT).

### Patient and public involvement

The conduct of the SHC originated from the knowledge of a high burden of injuries in youth handball and the Swedish Handball Federation was consulted in the planning and preparation of this study. Nutrition is key for health as well as sports performance and recovery and the dietary domains targeted in this study were determined based on clinical experience, involving active adolescents and practitioners to ensure relevant questions to the intended population. None of the above had any influence on the analyses, interpretation of the results or manuscript preparation.

## Results

Descriptive characteristics of the participants are presented in table 1. Forty-seven percent were female, and the mean age was 16.7±0.8 years and 16.7±0.9 years for males and females, respectively. The majority of the participants played handball on a regional level as backcourt players and went to first year of high school. Also, the majority of both males and females reported having had a previous knee or shoulder injury.

**Table 1:** Baseline participant characteristics presented as player seasons

	Male (n=908)	Female (n=795)
Age, mean years±SD	16.7±0.8	16.7±0.9
Playing handball, mean years±SD	9.4±2.3	9.8±2.0
School grade, n (%)		
1st year	360 (40)	289 (36)
2nd year	322 (35)	275 (35)
3rd year	226 (25)	231 (29)
Playing level past season, n (%)		
Regional level*	672 (74)	578 (73)
National level^†^	236 (26)	217 (27)
Playing position, n (%)		
Backcourt player	443 (49)	424 (53)
Wing player	198 (22)	147 (18)
Goalkeeper	133 (15)	123 (15)
Line player	134 (15)	101 (13)
Matches past season, mean n per week±SD	1.1±0.8	1.2±0.9
On-court training past season, mean hours per week±SD	6.5±2.4	6.3±2.3
Off-court training past season, mean hours per week±SD	4.2±2.2	4.1±2.0
Previous knee/shoulder injury, n (%)	497 (55)	434 (55)
Use of dietary supplements, n (%)	260 (29)	188 (24)

* Played for a local club or district team prior season.

† Played for a national youth team/summoned to a national camp prior season.

The proportion of participants with and without the potential risk dietary habit exposures at baseline is presented in table 2. Moderate-high NQ (ie, scoring ≥5 on the 12-point NQ index) was the most common dietary habit exposure in both males and females; whereas, low meal frequency (ie, <4 meals per day) was least common. It was more common to have one unfavourable dietary habit than ≥2 unfavourable dietary habits.

**Table 2:** Dietary habit exposure status at baseline

	Male (n=908)		Female (n=795)		Prevalence ratio^*^ (95% CI)
	Yes	No	Yes	No	
Low meal frequency†, n (%)	82 (9)	826 (91)	107 (13)	688 (87)	1.49 (1.14, 1.96)
Poor meal timing‡, n (%)	353 (39)	555 (61)	311 (39)	484 (61)	1.00 (0.88, 1.14)
Moderate-high NQ§, n (%)	432 (48)	476 (52)	499 (63)	296 (37)	1.32 (1.21, 1.44)
≥2 unfavourable dietary habits¶, n (%)	190 (21)	718 (79)	238 (30)	557 (70)	1.43 (1.22, 1.69)

* Prevalence ratio of dietary habit exposures comparing sex with male as reference group.

† <4 meals per day.

‡ No main meal within recommended time spans before/after training and no carbohydrate intake during matches.

§ ≥5 on the 12-point NQ index.

¶ <4 meals per day and/or having no main meal within recommended time spans before/after training and no carbohydrate intake during matches and/or scoring ≥5 on the 12-point NQ index.

NQnutritional quality

### Knee/shoulder injury incidence rate

Thirteen female player seasons (2%) were lost to follow-up, generating 782 female player seasons. A total of 199 events of knee/shoulder injuries occurred in females with a mean time at risk of 220 hours. Total time at risk was 79 328 hours, giving an incidence rate of 2.51 events/1000 hours of handball training and matches.

Fourteen male player seasons (2%) were lost to follow-up after baseline, generating a total sample of 894 male player seasons. A total of 173 events of knee/shoulder injuries occurred in males with a mean time at risk of 238 hours. Total time at risk for males was 87 721 hours, giving an incidence rate of 1.97 events/1000 hours of handball training and matches.

### Dietary habits, menstrual dysfunction and the association with injuries

**Figure 1:**
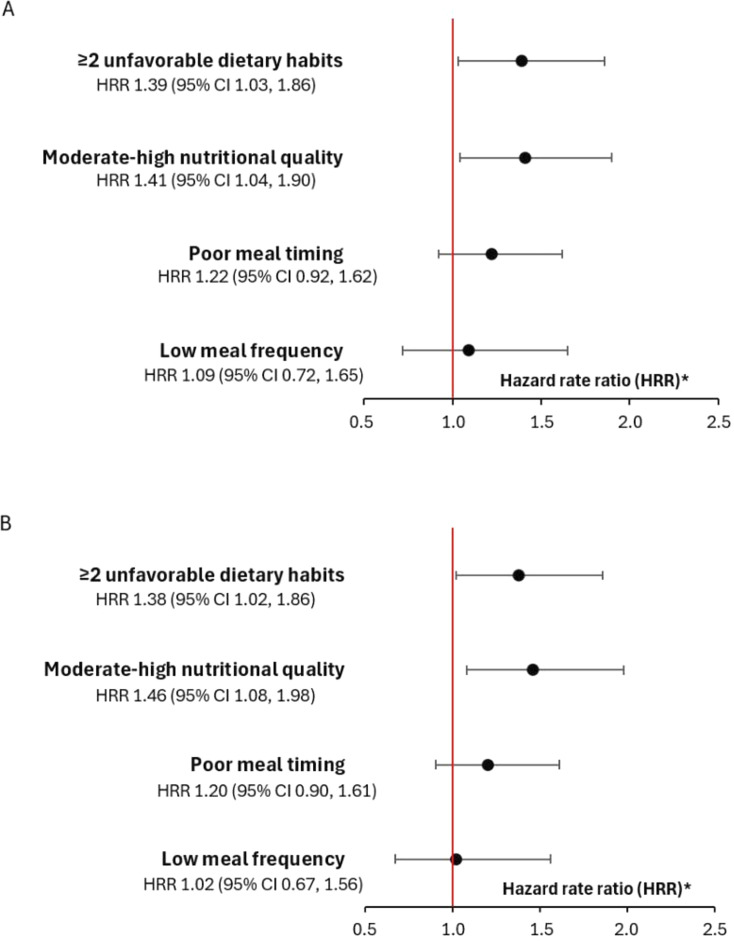
Hazard rate ratio with 95% CI of the (A) crude and (B) adjusted (previous knee/shoulder injury, playing position, playing level, use of dietary supplements and sleep hours) associations between dietary habits and knee/shoulder injury incidence in adolescent female handball players (n=782). ^*^Hazard rate ratio >1 indicates a higher injury risk, and <1 indicates a lower injury risk.

The crude and adjusted associations between dietary habits and knee/shoulder injury incidence in females are presented in figure 1. Crude Cox regression analyses generated an HRR for knee/shoulder injuries of 1.41 (95% CI 1.04, 1.90) for moderate-high NQ and adjusted analyses generated an HRR of 1.46 (95% CI 1.08, 1.98). For ≥2 unfavourable dietary habits, crude analyses generated an HRR of 1.39 (95% CI 1.03, 1.86) and adjusted analyses an HRR of 1.38 (95% CI 1.02, 1.86).

A total of 139 females (17%) reported no use of hormonal contraceptives at baseline in season 2022/2023 and were included in the analyses of the association between menstrual dysfunction and injuries. Of these, 33 (24%) had a menstrual dysfunction. The crude and adjusted analyses generated an HRR for menstrual dysfunction of 0.75 (95% CI 0.36, 1.56) and 1.03 (95% CI 0.45, 2.36), respectively.

**Figure 2:**
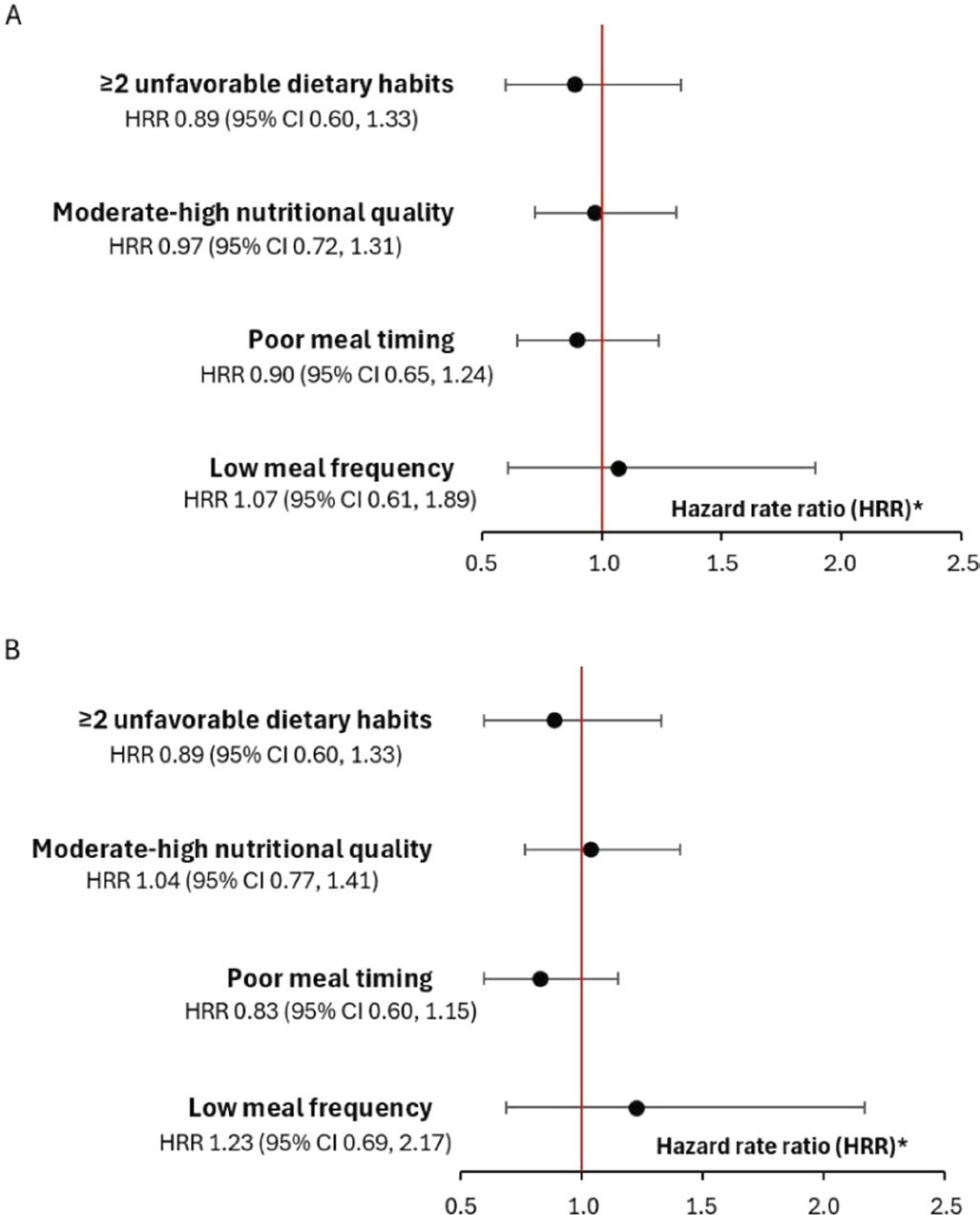
Hazard rate ratio with 95% CI of the (A) crude and (B) adjusted (previous knee/shoulder injury, playing position, playing level, use of dietary supplements and sleep hours) associations between dietary habits and knee/shoulder injury incidence in adolescent male handball players (n=894). ^*^Hazard rate ratio >1 indicates a higher injury risk, and <1 indicates a lower injury risk.

Crude and adjusted HRR for the associations between dietary habits and injuries in males are presented in figure 2. Crude Cox regression analyses generated an HRR for knee/shoulder injuries of 1.07 (95% CI 0.61, 1.89) for low meal frequency and adjusted analyses generated an HRR of 1.23 (95% CI 0.69, 2.17). For poor meal timing, crude analyses generated an HRR of 0.90 (95% CI 0.65, 1.24) and adjusted analyses an HRR of 0.83 (95% CI 0.60, 1.15).

Sensitivity analyses for each exposure of dietary habits and the outcome, with adjustment for being included more than one season, are presented in online supplemental table 3. Overall, inclusion in more than one season did not change the associations.

## Discussion

### Dietary habits and the association with injuries

The main findings of this study were that a moderate-high NQ and ≥2 unfavourable dietary habits (not meeting the sports nutrition recommendations meal frequency, meal timing or having a moderate-high NQ) were associated with a 46% and 38% higher injury incidence in female adolescent handball players, respectively, compared with low NQ and only one unfavourable dietary habit. The motivation for including a variable with ≥2 unfavourable dietary habits was to capture a potential additional effect by having more than one dietary habit not according to recommendations. For example, participants with a low meal frequency may have the chance to meet their daily energy requirements even with fewer energy-dense meals than the recommended 4–6 daily meals. However, participants with a low meal frequency who have a moderate or high NQ (ie, likely an overall less energy-dense diet) might have more challenges with meeting the energy requirements, hence, be at higher risk of problematic LEA. Given that this study found an association between ≥2 unfavourable dietary habits and injuries, this is a possible explanation. Furthermore, poor meal timing was associated with a 20% increased injury incidence in females, but the results were unprecise given the wide CI including the null association. Also, no associations were found between low meal frequency and injuries, which contradicts a previous study in adolescent female skaters where meal skipping was associated with injuries.^20^

The associations between low meal frequency and poor meal timing, respectively, and injuries in male adolescent handball players, were unprecise with a wide CI including the null association. Also, no associations were found for moderate-high NQ in males. This could be explained by this group of male athletes having sufficient energy intakes in relation to their demands or due to misreporting of dietary habits. However, it should be emphasised that LEA, as well as disordered eating and eating disorders, are prevalent among male team-sport athletes,^30^ and more studies assessing REDs in males and the potential impacts on health and performance are needed.^31^ Also, future studies are warranted that investigate the potential sex differences in diet-energy balance in team sports to further deepen the understanding of dietary habits and injuries in males.

This study found that a moderate-high NQ (ie, moderate-high adherence to the NNR) was a potential injury risk factor in females. The NNR aim to promote healthy eating in the general population to prevent lifestyle-related conditions of public health concern such as obesity, type 2 diabetes and cardiovascular diseases. Therefore, the NNR include a high intake of nutrient-dense foods (eg, fruits/vegetables, fish, whole grains, fibre) and low intake of energy-dense foods with less nutrients (eg, refined grains, fast foods, candy and sugar-sweetened beverages). Although adhering to the NNR increases the chance of meeting requirements of essential micronutrients in athletes as well as non-athletes, the general recommendations are less suitable for athletes.^7 32^ There are several challenges associated with meeting the extensive energy and carbohydrate requirements as an adolescent athlete, including exercise-induced appetite-suppression and limited food availability^32^ as well as restrictive eating behaviours.^33^ Adolescent athletes are recommended to have a sufficient intake of carbohydrates and energy-dense foods,^7 32^ yet female field-based team sports athletes tend to have insufficient intakes of energy as well as carbohydrates.^34^ A low energy-dense diet high in fibre, as promoted through the NNR, has been associated with problematic LEA in female athletes.^35^ Our cross-sectional study^22^ in adolescent handball players, with a population included also in the current study, found a tendency towards restrictive dietary habits in females, which might explain the association between a moderate-high NQ and injuries in the current study.

### Menstrual dysfunction and the association with injuries in females

Only a small proportion (17%) of the females reported no use of hormonal contraceptives at baseline and were included in the analyses. The prevalence of menstrual dysfunction of 24% is what would be expected compared with previous reports of female athletes.^9 36^ No associations were found with injuries with a wide CI including the null association, in contrast with previous findings.^11^,^13^

Several possible reasons exist to explain the conflicting findings. First, only 33 females were included in the Cox regression analyses. It is furthermore possible that menstrual dysfunction in the included females could be due to other reasons than LEA, such as polycystic ovary syndrome or not having established regular menstrual bleeding due to young age,^27^ or that a potential underlying LEA was not long-term/severe but could have impacted injury risk if followed for a longer period of time. Alternatively, there might be no association or associations between menstrual dysfunction and other injury types.

### Limitations

This study includes a large sample of adolescent handball players from the majority of the handball-profiled high schools in Sweden with a high follow-up rate, which reduces the risk of selection bias. Also, a cohort without the outcome at baseline strengthens the validity of a causal association. Yet, residual or unmeasured confounding cannot be ruled out, and ascertaining causality is therefore not possible. As an example, it is possible that non-reported injuries in other body areas than knee and shoulders could have had an impact on the choice of dietary habits prior to enrolment in the study. Moreover, measures of other potential confounders that might explain the found associations, such as psychosocial factors, are lacking, and should suggestively be included in future studies. The rationale for including different measures of dietary habits was based on the fact that self-registered methods for ascertaining energy and nutrient intakes (eg, weighed food record) are not feasible in large observational studies. However, self-reported health behaviours tend to be reported in accordance with social desirability. Using questions about dietary habits validated in adolescent athletes would possibly reduce the risk of misclassification of the exposures. Menstrual dysfunction was measured as a proxy for problematic LEA, yet collected only during one season, which likely could introduce a sparse-data bias considering the findings with relatively wide CIs. Evaluating these symptoms for problematic LEA during several seasons would yield more comprehensive insights. Since the SHC is ongoing and a new cohort of adolescent handball players is included every season, we will be able to use extensive menstruation data across multiple seasons in the future to assess its relationship with injuries. Also, the lack of measurements of eating attitudes and other symptoms of REDs in females (eg, mental problems) as well as in males (eg, libido) is a limitation.

The injury assessment was conducted based on best-practice recommendations^37^ including the widely adopted OSTRC-O for capturing gradual and sudden onset injuries, where impact on athletes’ participation and sporting performance defined the severity of the problem (ie, substantial injury). Instead, having asked about injuries without accounting for impact on training/performance would likely have biased the results since many athletes train despite injuries and pain.^37^ Yet, if the association between dietary habits and injuries differs between injury types, the results may not be generalisable. A substantial problem captured based on impact on training and performance might introduce random errors such as choosing the wrong answer in the survey. Also, the OSTRC-O captures a problem but does not assess what type of problem (eg, pain, stiffness). To yield more nuanced insights about injuries, future studies using the OSTRC-O could consider also stratifying injuries by type or severity.

This study included participants throughout three seasons and 41% of the participants were enrolled in more than one season. Sensitivity analyses with adjustment for being included in the SHC more than one season were performed, however, did not change the associations. It is also reasonable to assume that enrolment in several seasons is not associated with dietary habits.

## Conclusions

In adolescent female handball players, a moderate-high nutritional quality and having more than one unfavourable dietary habit are associated with higher knee/shoulder injury incidence while no or unprecise associations were found for meal frequency, meal timing and menstrual dysfunction, as well as for male adolescent handball players. This study sheds light on the potential importance of promotion of sports nutrition recommendations for athletes for a healthy athletic career.

## supplementary material

10.1136/bmjsem-2024-002332 online supplemental file 1

## Data Availability

Data are available upon reasonable request.
