# Supplementary material for: Dietary habits and knee and shoulder injury incidence in adolescent male and female handball players: the Swedish Handball Cohort
Source: BMJ Open Sport Exerc Med. 2025 Mar 23;11(1):e002332. doi: 10.1136/bmjsem-2024-002332 (PMC11934375; doi:10.1136/bmjsem-2024-002332)
Supplement: online supplemental file 1 [file bmjsem-11-1-s001.docx]

**Supplemental table 1**. Summary of exposures, covariates, and outcomes assessment and categorization in statistical analyses.

| **BASELINE: EXPOSURES** | | | | |
| --- | --- | --- | --- | --- |
|  | | Question | Answer options | Categorization in statistical analyses |
| **Meal frequency** | | How many days/week do you eat...  .. Breakfast?  .. Morning snack?  .. Lunch?  .. Afternoon snack?  .. Dinner?  .. Evening snack?  .. Other meal (1)?  .. Other meal (2)? | 0-7 days per week | Binary (1,0): <4 meals per day |
| **Meal timing** | |  |  |  |
| Before training | Do you usually eat within 3 hours before your...  .. Morning training?  .. Evening training? | Main meal before training  Snack before training  No meal before training | Binary (1,0): no main meal neither 3 hours before nor within 1 hour after morning and evening training, respectively, and no carbohydrate intake during matches |  |
| After training | Do you usually eat within 1 hour after your..  .. Morning training?  .. Evening training? | Main meal after training  Snack after training  No meal after training |  |  |
| During training | | Do you consume sport drinks (mix of water, sugar and salt – not energy drink), gainers, gels or bars during training/matches? | Yes  No |  |
| **Nutritional quality** | | How often do you eat…  .. Fruits?  .. Vegetables?  .. Whole-grain bread?  .. Crisp bread?  .. Fried potatoes/French fries?  .. Fish/seafood?  .. Sausage?  .. Chocolate/candy?  .. Pastries?  .. High-fat cheese?  .. Sugar-sweetened beverages? | Never or <1 time per month  1-3 times per month  1-6 times per week  1-3 times per day  4 times per day or more | Binary (1,0): nutritional quality score ≥5 of 12, corresponding to moderate or high nutritional quality |
| **Menstrual function** | |  |  |  |
| Contraceptives | Are you using hormonal contraceptives such as pills, intrauterine device, patch, ring, implant, or injection? | Yes  No | Binary (1,0): primary amenorrhea >15 years of age, ≥3 months since last menstruation or ≤8 menstruations past 12 months in females reporting no use of hormonal contraceptives |  |
| Last period | When was the last time you had your period? | 0-4 weeks 1-2 months 3-4 months >5 months  No first period |  |  |
| Periods past year | How many times have you gotten your period during the past year? | 12 or more times 9-11 times 6-8 times 3-5 times 0-2 times |  |  |
| **BASELINE: COVARIATES** | | | | |
|  | | Question | Answer options | Categorization in statistical analyses |
| **Sleep hours** | | How many hours/night do you sleep during..  .. Weekdays?  .. Weekends? | 0-24 hours | Continuous: mean sleep hours during weekdays and weekends |
| **Playing position** | | Which playing position do you have? | Goalkeeper  Wing player  Line player  Backcourt player | Categorical: each position separately |
| **Playing level** | | Which playing level do you have? | Regional level  National level | Binary (1,0): regional level |
| **Use of dietary supplements** | | Do you use any dietary supplements (e.g., vitamins, minerals etc.)? | Yes No | Binary (1,0): use of dietary supplements |
| **Previous injury** | |  |  |  |
| Shoulder | Have you had a previous shoulder injury during your handball career? | Yes No | Binary (1,0): a knee or shoulder injury during the handball career |  |
| Knee | Have you had a previous knee injury during your handball career? | Yes  No |  |  |
| **WEEKLY FOLLOW-UPS: OUTCOME** | | | | |
|  | | Question | Answer options | Categorization in statistical analyses |
| **Substantial shoulder problem** | |  |  |  |
| Gatekeeper question | | Have you had any difficulties in participating in your sport (usual training/match/competition) due to a shoulder problem the past week? | Full participation, without shoulder problem  Full participation, with shoulder problem  Reduced participation due to shoulder problem  Could not participate due to shoulder problem  Could not participate/reduced participation due to other reason than a shoulder problem | Participants responding other than Full participation, without shoulder problem received the questions below about reduced training and reduced performance |
| Reduced training | To what extent have you reduced your training volume due to shoulder problems during the past week? | No reduction  To a minor extent  To a moderate extent  To a major extent  No participation at all due to shoulder problems | Binary (1,0): moderate reduction, major reduction, or no participation at all due to shoulder problems regarding reduced training or performance (defined as a shoulder injury) |  |
| Reduced performance | To what extent have shoulder problems affected your performance during the past week? | No effect  To a minor extent  To a moderate extent  To a major extent  No participation at all due to shoulder problems |  |  |
| **Substantial knee problem** | |  |  |  |
| Gatekeeper question | | Have you had any difficulties in participating in your sport (usual training/match/competition) due to a knee problem the past week? | Full participation, without knee problem  Full participation, with knee problem  Reduced participation due to knee problem  Could not participate due to knee problem  Could not participate/reduced participation due to other reason than a knee problem | Participants responding other than Full participation, without shoulder problem received the questions below about reduced training and reduced performance |
| Reduced training | To what extent have you reduced your training volume due to knee problems during the past week? | No reduction  To a minor extent  To a moderate extent  To a major extent  No participation at all due to knee problems | Binary (1,0): moderate reduction, major reduction, or no participation at all due to knee problems regarding reduced training or performance (defined as a knee injury) |  |
| Reduced performance | To what extent have knee problems affected your performance during the past week? | No effect  To a minor extent  To a moderate extent  To a major extent  No participation at all due to knee problems |  |  |
| **WEEKLY FOLLOW-UPS: TIME AT RISK** | | | |  |
|  | Question | Answer options | Categorization in statistical analyses |  |
| **Playing handball** |  |  |  |  |
| Handball training | How many hours have you in total trained handball on court during the past week? | Free text | Continuous total minutes of handball training and handball matches past week |  |
| Handball matches | How many minutes of handball matches have you played during the past week? | Free text |  |  |

**Supplemental table 2**. Scoring of food frequency intake for assessing nutritional quality (score 0-12).

|  | Unit | 0 points | 1 point | 2 points |
| --- | --- | --- | --- | --- |
| Fruits/vegetables | Times per day | <3 | 3-4 | ≥5 |
| Bread (whole-grain and crisp) | Slices per day | <1 | 1-2 | ≥3 |
| Fish/seafood | Times per week | <1 | 1-2 | >2 |
| Candy, pastries, sugar-sweetened drinks, French fries | Times per week | ≥7 | 3-6 | <3 |
| Dietary fat on sandwich | Fat content | ≥60% | ≤40% |  |
| Cheese, high fat | Times per week | ≥4 | 2-3 | ≤1 |
| Sausage | Times per week | >1 | ≤1 |  |


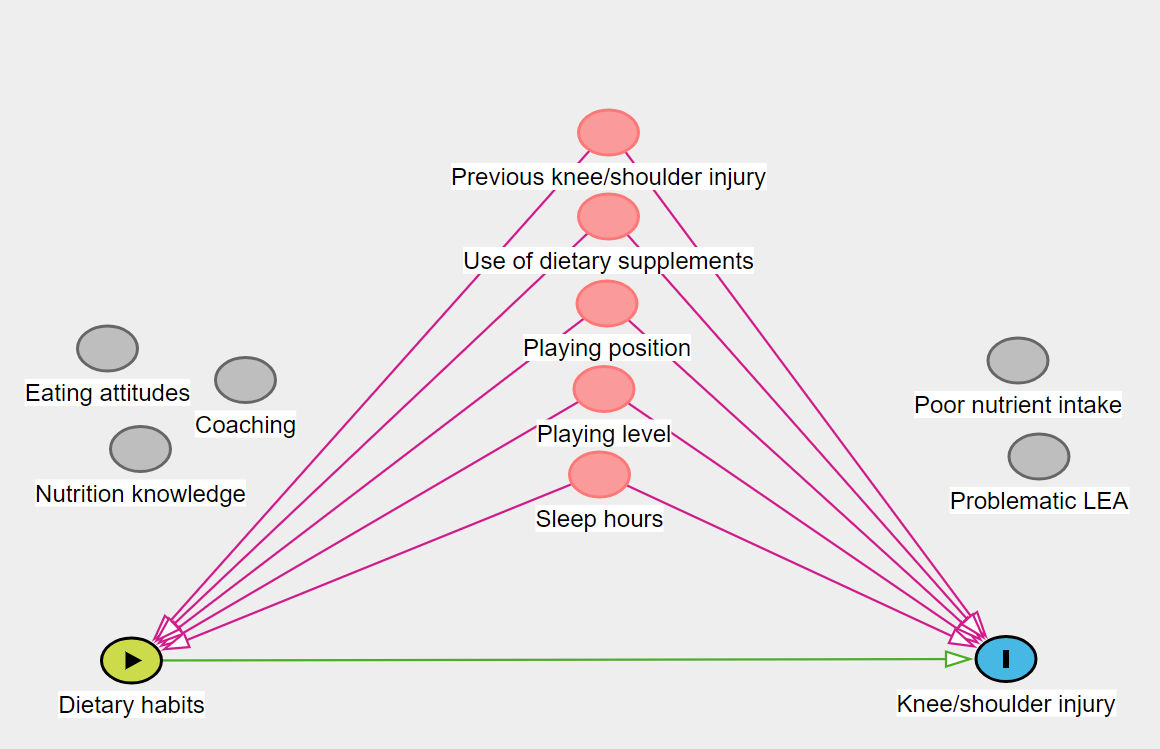


**Supplemental figure 1.** Directed acyclic graph visualizing the association between dietary habits and knee/shoulder injury incidence with potential confounders. Gray circles indicate variables important for the mechanism in the potential association but is not measured in the Swedish Handball Cohort. Abbreviations: LEA, low energy availability.

**Supplemental table 3.** Sensitivity analyses with hazard rate ratio (HRR) and 95% confidence interval (CI) of the associations between dietary habits and knee/shoulder injury incidence, with adjustment for being included in the Swedish Handball Cohort more than one season.

|  | Male (n=894) | Female (n=782) |
| --- | --- | --- |
| Low meal frequency^1^ | HRR 1.08 (95% CI 0.61, 1.91) | HRR 1.05 (95% CI 0.69, 1.58) |
| Poor meal timing^2^ | HRR 0.89 (95% CI 0.64, 1.22) | HRR 1.23 (95% CI 0.92, 1.63) |
| Moderate-high NQ^3^ | HRR 0.95 (95% CI 0.70, 1.28) | HRR 1.45 (95% CI 1.07, 1.96) |
| ≥2 unfavorable dietary habits^4^ | HRR 0.87 (95% CI 0.58, 1.29) | HRR 1.42 (95% CI 1.06, 1.91) |

Abbreviations: NQ, nutritional quality
^1^<4 meals/day.
^2^No main meal within recommended time spans before/after training and no carbohydrate intake during matches.
^3^≥5 on the 12-point NQ index.
^4^<4 meals/day or having no main meal within recommended time spans before/after training and no carbohydrate intake during matches or scoring ≥5 on the 12-point NQ index.
